# Utility of video-assisted method for identifying and preserving the external branch of the superior laryngeal nerve during thyroidectomy

**DOI:** 10.3389/fsurg.2023.1118083

**Published:** 2023-04-18

**Authors:** Zhaoming Ding, Ruinan Sheng, Liang Zhang, Jihua Han, Mengshi Chen, Wen Bi, Xuesong Zhao, Jiewu Zhang, Chunlei Nie

**Affiliations:** Department of Thyroid Surgery, Harbin Medical University Cancer Hospital, Harbin, China

**Keywords:** EBSLN, thyroidectomy, neuroanatomy, anatomical variations, cernea classification, surgical techniques

## Abstract

**Background:**

The EBSLN is vulnerable to damage during thyroidectomy, results in voice related complications, negatively affect patient quality of life, routine identification of the EBSLN prior to surgical manipulation is necessary for a complication-free thyroidectomy. We aimed to validate a video-assisted procedure for identifying and preserving the external branch of the superior laryngeal nerve (EBSLN) during thyroidectomy and analyze the EBSLN Cernea classification and the localization of the nerve entry point (NEP) from the insertion of the sternothyroid muscle.

**Methods:**

A prospective descriptive study was performed; 134 patients, who scheduled for lobectomy with an intraglandular tumor max diameter ≤ 4 cm and without extrathyroidal extension, were randomly divided into the video-assisted surgery (VAS) and conventional open surgery (COS) groups. We used the video-assisted surgical procedure for visually identifying the EBSLN directly, and compared the differences in the visual identification rate and total identification rate of the two groups. We also measured the localization of the NEP using the insertion of the sternothyroid muscle as a reference.

**Results:**

There was no statistically significant difference in clinical characteristics between the two groups. The visual identification rate and total identification rate were significantly higher in the VAS group than the COS group (91.04% vs. 77.61%, 100% vs. 89.6%). The EBSLN injury rate was 0 in both groups. The mean vertical distance (VD) of the NEP from the sternal thyroid insertion was 1.18 mm (SD 1.12 mm, range, 0–5 mm), with approximately 88.97% of the results occurring within the 0–2 mm range. The mean horizontal distance (HD) was 9.33 mm (SD 5.03 mm, range, 0–30 mm), with over 92.13% of the results occurring within the 5–15 mm range.

**Conclusion:**

Both the visual and total identification rates of the EBSLN were significantly higher in the VAS group. This method provided a good visual exposure rate of the EBSLN, aiding in identifying and protecting the EBSLN during thyroidectomy.

## 1. Introduction

The external branch of the superior laryngeal nerve (EBSLN) is an important nerve tissue in thyroid surgery (1, 2), similar to the recurrent laryngeal nerve (RLN) and the parathyroid gland, and is responsible for motor innervation of the cricothyroid muscle (CTM), which acts to elongate and thin the true vocal folds during phonation, thereby elevating the pitch of the voice. Dysfunction of the CTM secondary to EBSLN injury results in hoarseness, a weak voice, vocal fatigue, or reduced vocal frequency; these consequences may be permanent and negatively affect patient quality of life, especially for professional voice users (3, 4).

Considering its high injury rate (up to 58%) and varying exposure rate (5, 6), routine identification of the EBSLN prior to surgical manipulation is necessary for a complication-free thyroidectomy (7, 8). The EBSLN descends posterior to the sternothyroid muscle towards the cricothyroid muscles, along and cross the STA. As described by Cernea classification, types 1, 2A and 2B means more than 1 cm above the upper pole of thyroid, within 1 cm of the upper pole, or below the upper pole respectively. While there are techniques to identify the RLN during thyroid surgery, there is no clear consensus or standard technique for the identification of the EBSLN. Due to its close anatomical relationship with the superior thyroid artery, its fine structure, and its highly variable anatomy (9), its susceptibility to injury and the difficulty in exposing the EBSLN during thyroidectomy are well recognized (10). Inadequate exposure of the upper polar region is the major factor affecting EBSLN identification and iatrogenic injury, which is mainly caused by occlusion of the sternalthyroid muscle, which is also assessed as a landmark for the nerve entry point (NEP) of the EBSLN on the inferior constrictor muscle (11, 12).

The aim of the present study was to validate a video-assisted method for visually identifying and preserving the EBSLN accurately and feasibly by expanding the scope and eliminating the dead angle of observation.

## 2. Methods

### 2.1. Study design

This was a descriptive study of a prospective case series of conventional open thyroidectomy performed from February 2021 to July 2022 in our department.

The inclusion criteria included the following: (1) adult patients who were scheduled for lobectomy, according to the standard of the ATA Thyroid Guidelines (13); (2) patients with an intraglandular tumor max diameter ≤ 4 cm; and (3) patients without extrathyroidal extension. The exclusion criteria included (1) previous thyroid or neck surgery, (2) ipsilateral neck dissection, (3) neck irradiation, (4) pregnant woman, and (5) preoperative vocal fold palsy.

All surgeries were performed by the same surgical team. This study was approved by the ethics committee of our hospital, and written informed consent was obtained from all patients.

### 2.2. Surgery

All patients underwent open thyroid lobectomy under general anesthesia by the same surgical team. To ensure the full return of muscular activity, a minimal single dose of a nondepolarizing muscle relaxant (rocuronium, approximately 0.5 mg/kg) was used, as previous described (14).

In the video-assisted surgery (VAS) group, a transverse low-collar skin incision was made, platysmal flaps were dissociated and raised, and the thyroid was exposed with a longitudinal incision in the linea alba cervicalis. The sternohyoid and sternothyroid muscles were retracted upward and laterally, while the superior pole of the thyroid lobe was retracted inferolaterally, and an avascular window was opened in the sternothyroid–laryngeal triangle, which constituted the sternothyroid muscle, the inferior constrictor muscle of the pharynx and the cricothyroid muscle, and the thyroid.

**Figure 1:**
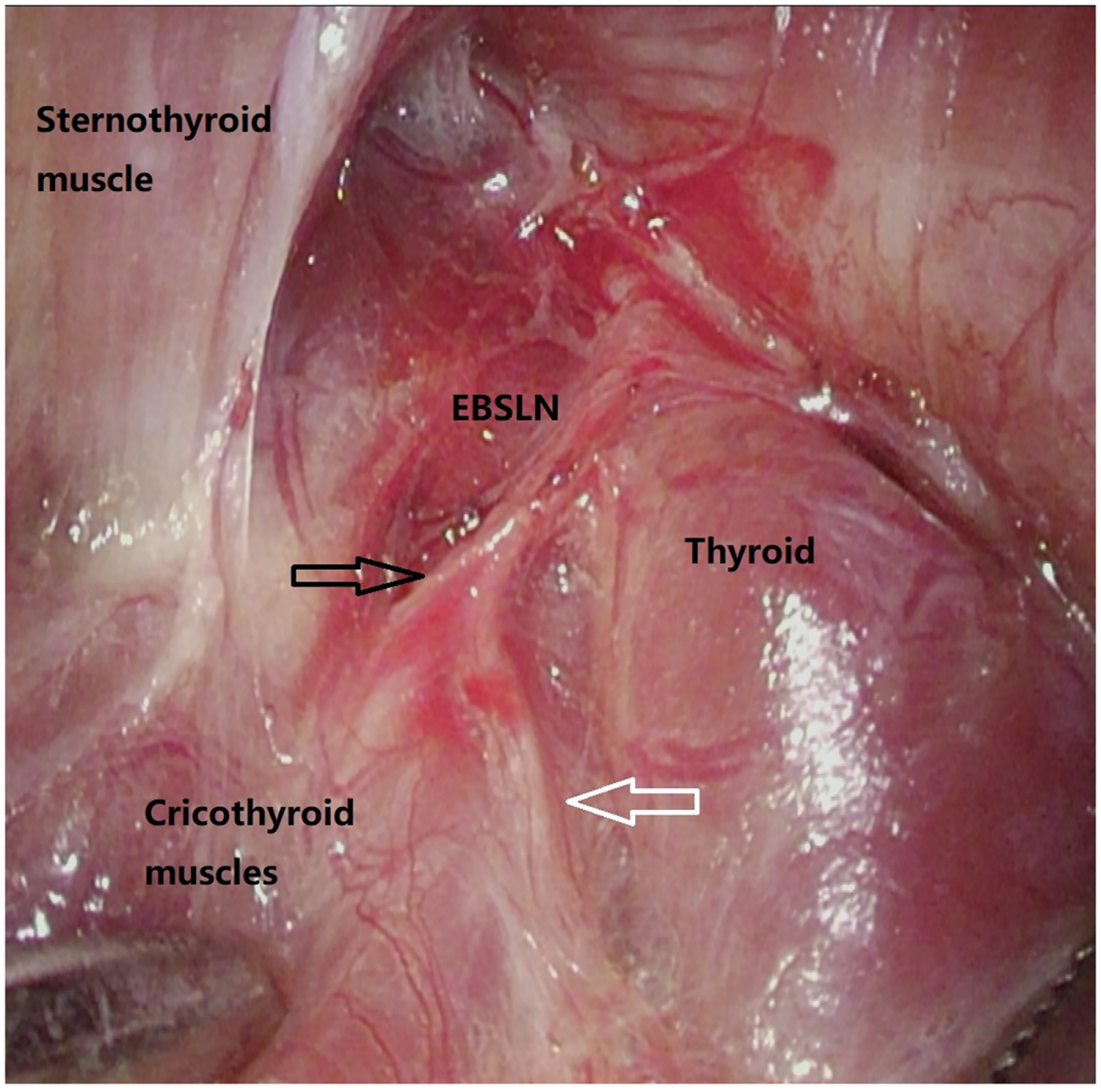
Visually expose of EBSLN by video-assisted view angle. Black arrow: EBSLN toward cricothyroid muscles, white arrow: communicating branch between EBSLN and RLN.

Through video assistance, combining blunt separation and meticulous dissection, we attempted to visually identify the EBSLN in this area (Figure 1) and confirmed it with a nerve stimulator. Contraction of the CTM under electrostimulation of 1 mA represents the functional integrity of the EBSLN during operation (14).

After identification, the exposed nerve was followed downstream until it entered the inferior constrictor or cricothyroid muscle at the nerve entry point (NEP) and traced upstream until it crossed over the STA. If the nerve was not clearly visualized, then the nerve tract on the constrictor muscle was indirectly stimulated to create and observe the physiological response of the CTM. If the nerve was still not identified, the search for the nerve was discontinued to avoid excessive damage caused by excessive dissociation, and the superior thyroid vessels were adequately dissected for safe ligation and division.

In the conventional open surgery (COS) group, the main step was similar but involved only the use of the naked eye without the assistance of a laparoscope.

### 2.3. Outcome measurements

We studied the following aspects: (1) the rate of EBSLN identification wherein the total identification rate included both the visual identification rate of the EBSLN confirmed by intraoperative nerve monitoring (IONM) and the functional identification rate of nonvisualized EBSLNs confirmed by IONM; (2) the rate of EBSLN injury for which we evaluated EBSLN injury by (1) anatomical or functional integrity damage during the operation, (2). positive findings on postoperative laryngoscopy, including posterior glottis rotation toward the affected side, inferior vocal fold positioning on the paralyzed side, and epiglottic petiole deviation toward the affected side (15, 16), and (3) voice assessment, including maximum phonation time (MPT, sec), voice level (VL, dB), and fundamental frequency (Fo, Hz), which were measured as previously described (17), and the quality of voice, which was measured using the VoiSS questionnaire focused on patient perspectives (18); (3) the percentage distribution of the Cernea classification, which describes the anatomic variations in the EBSLN in relation to the intersection of the superior thyroid artery and superior thyroid pole (19); (4) the localization of the NEP, which is defined by two distances (11), namely, the vertical distance (VD), which is the perpendicular distance from the NEP to the sternothyroid insertion onto the oblique line of the thyroid cartilage, and the horizontal distance (HD), which is the distance from the NEP to the anterior border of the sternothyroid muscle; and (5) the operative time from the incision of the skin to dissection of the upper pole of the thyroid area.

### 2.4. Statistical analysis

Data are expressed as the mean ± standard deviation (SD) and were analyzed with SPSS 22.0 statistical software (SPSS Inc., Chicago, IL, USA). The statistical significance of categorical variables was evaluated by the chi-square (*χ*^2^) test or Fisher's exact test. *P* < 0.05 was considered to indicate significance.

## 3. Results

### 3.1. Characteristics of the study population

A total of 134 patients qualified for inclusion in the study. All operations were completed smoothly. A total of 67 patients were enrolled in the VAS group, and their mean age was 45.37 ± 9.792 years (range, 20–68). A total of 67 patients were enrolled in the COS group, and their mean age was 43.96 ± 9.516 years (range, 24–62). The general distribution of the patients is shown in Table 1.

**Table 1:** Characteristics of the enrolled patients (*n* = 134).

Characteristics	Video assisted surgery group (*n* = 67)		Convertional open surgery group (*n* = 67)		*P*-Value
	No.	%	No.	%	
Case	67		67		
Age					
X ≤ 55	58	86.57	57	85.07	0.804
X > 55	9	13.43	10	14.93	
Mean ± SD	45.37 ± 9.792		43.96 ± 9.516		0.397
Sex					
Male	15	22.39	14	20.895	0.834
Female	52	77.61	53	79.104	
Side					0.604
Left	30	44.78	33	49.25	
Right	37	55.22	34	5.075	
Tumor location					
Upper	14	20.895	13	19.4	0.86
Middle	32	47.76	30	44.78	
Lower	21	31.34	24	35.82	
Tumor size					
≤1 cm	32	47.76	30	44.78	0.928
1–2 cm	28	41.79	29	43.28	
2–4 cm	7	10.45	8	11.94	
Pathologic diagnosis					
PTC	67	100	67	100	1

### 3.2. Identification of the EBSLN

We first tried to visually identify the EBSLN during upper pole dissection. Visual identification rates were 91.04% (61 in 67 cases) and 77.61% (52 in 67 cases) in the VAS group and the COS group, respectively (*P* < 0.05). Electrophysiological stimulation of all visually identified nerve branches generated CTM contractions that showed functional integrity. In addition to visual identification, nonvisualized nerve branches were functionally discovered in 6 and 8 nerves by IONM in the VAS group and the COS group, respectively. In total, in the VAS group, 67 (100%) EBSLNs were identified, and in the COS group, 60 (89.6%) EBSLNs were identified (*P* < 0.05) (Table 2).

**Table 2:** Identification rate of the EBSLN in the VAS group and COS group.

Identification of the EBSLN	Video-assisted surgery group (*n* = 67)		Conventional open surgery group (*n* = 67)		*P*-Value
	No.	%	No.	%	
Visual identification and confirmed by IONM	61	91.04	52	77.61	0.032
Nonvisualized but identified by IONM additionally	6	8.96	8	11.94	
Total identification	67	100	60	89.55	0.020

### 3.3. Injury rate of the EBSLN

The injury rate of the EBSLN was 0 in both groups, with no significant differences. All EBSLNs identified during the operation were confirmed to have anatomical and functional integrity before the end of the operation. All patients underwent postoperative stroboscopic laryngoscopy, and we did not find any signs of EBSLN injury. On functional voice assessment, a 10% or higher decrease in phonation parameters (MPT, VL, and Fo) was not found in any patients in the two groups. The postoperative VoiSS questionnaire scores of all patients were within the normal range, and there was no significant difference between the two groups (Table 3).

**Table 3:** Comparison of EBSLN injury and postoperative functional voice assessment.

Variable	Video-assisted surgery group (*n* = 67)	Conventional open surgery group (*n* = 67)	*P*
EBSLN injury observed during operation			
Interruption of anatomical integrity	0	0	
Damage of functional integrity	0	0	
Decrease >10% from preop baseline			
MPT	0	0	
VL (dB)	0	0	
Fo (Hz)	0	0	
VoiSS score	10.78 ± 1.686	11.04 ± 1.471	0.328

### 3.4. Classification of the EBSLN and localization of the NEP

In the VAS group, all 67 EBSLNs were identified, among these, 9 (13.43%) were Cernea type 1, 26 (38.81%) were type 2A, and 32 (47.76%) were type 2B. while in the VAS group, 60 EBSLNs were identified, 5 (8.33%) were Cernea type 1, 24 (40.0%) were type 2A, and 31 (51.67%) were type 2B. There is no statistical difference in the distribution of Cernea type between the two groups (Table 4).

**Table 4:** Distribution of identified EBSLN according to cernea classification.

	Video assisted surgery group (*n* = 67)		Conventional open surgery group (*n* = 60)		*P*-Value
	No.	%	No.	%	
TYPE 1	9	13.43	5	8.33	0.652
TYPE 2A	26	38.81	24	40.00	
TYPE 2B	32	47.76	31	51.67	

Among the total of 127 EBSLNs identified in the two groups, 14 (11.02%) were Cernea type 1, 50 (39.37%) were type 2A, and 63 (49.61%) were type 2B.

We also analyzed the localization of the NEP based on the distances from the sternothyroid insertion. The mean vertical distance (VD) was 1.18 mm (SD 1.12 mm, range, 0–5 mm), with approximately 88.97% occurring within the 0–2 mm range, and the mean horizontal distance (HD) was 9.33 mm (SD 5.03 mm, range, 0–30 mm), with over 92.13% occurring within the 5–15 mm range.

### 3.5. Comparison of the operative time

The average time from skin incision to the dissection of the upper pole was 15.90 ± 3.802 min and 16.36 ± 4.191 min in the VAS group and the COS group, respectively, and there were no significant differences between the two groups.

## 4. Discussion

The fine size, variable course and label of “the neglected nerve” make the EBSLN at potential risk during thyroidectomy (20, 21). Injury to the EBSLN can occur by transection, traction, ligation, thermal damage, and disrupted blood supply (22), and identification though proper and sufficient exposure is important to avoid this (8). The laryngeal head of the sternothyroid muscle is assessed as a landmark for the course of the EBSLN on the inferior constrictor, but it also obstructs the surgeon's observation of the superior pole of the thyroid region and affects the exposure of the EBSLN (11, 23).

Some surgeons used to cut off part of the laryngeal head of the sternothyroid muscle to better expose the EBSLN (24). This process not only increases muscle damage but also increases the chance of unintentional EBSLN injury. To avoid this, a strap intermuscular approach has been described for exposing and protecting the EBSLN (25). Considering that the main factor affecting the exposure of the upper polar region is the angle of view, not the sternal thyroid muscle itself, we tried to change the visual angle and afford sufficient visualization of the superior thyroid region by means of endoscopic assistance, without sacrificing the integrity of the sternothyroid muscle or an extra expanded separation area.

Compared to COS, the new video-assisted method does not require amputation of the sternothyroid muscle, provides a better visual field to expose the EBSLN, and makes it more easily protected intraoperatively. Additionally, the new video-assisted method does not increase the time from cutting the skin to exposing the upper thyroid region. This method not only uses the magnifying effect of the endoscope to expand the observation field but also reduces surgical injury through precise and directional operation.

Currently, IONM has gained extensive acceptance as an adjunct to the gold standard of EBSLN identification (26). For the nerves that cannot be identified accurately and those that run inside the pharyngeal constrictor muscle (Friedman's classification type 3 (27), IONM plays an important role in EBSLN protection (2, 28). Moreover, visual exposure and protection can confirm the anatomical integrity of nerves, but functional integrity cannot be confirmed. Before the end of the operation, while anatomical integrity is confirmed, neural monitoring is required to exclude the elimination of thermal damage and strain injury and verify neural function. Electrostimulation causes simultaneous contraction of the cricothyroid muscle in both the straight and oblique abdomen as the standard of neurological integrity (3, 14, 29).

Nevertheless, IONM does not completely eliminate the risk of nerve injury caused by unintentional injuries (30, 31), and the surgeon's experience and full understanding of anatomy and possible variations are more important than the aid of high-tech instruments during thyroid surgery (32). Therefore, our strategy mainly depends on visual confirmation of the nerve during surgery, and electrical nerve stimulation is used for confirmation after exposure and functional examination before the end of the surgery.

In the present study, we achieved a 91% EBSLN exposure rate (61 in 67 nerves) based on visual exposure and confirmed that nerve function was good through the contraction of the cricothyroid muscle. This rate is satisfactory compared with those reported in previous studies with IONM (33–35).

We used IONM to further increase the exposure rate by 9% (6 in 67 nerves), and the total exposure rate of the EBSLN reached 100%. This result revealed that the EBSLN could be visually identified in the majority of cases, with or without IONM. For hospitals without neural monitoring equipment or the economic ability to purchase neural monitoring equipment, medical insurance limits expenses, and this method is conducive to the identification and isolation of the nerve, thereby minimizing iatrogenic injury and postoperative complications.

In the present study, we also calculated the distribution of Cernea groupings of the EBSLNs. Among the total of 127 EBSLNs identified in the two groups, we found a high prevalence of Cernea type 2 nerves (88.98%), with 49.61% being type 2B. These data are similar to those reported in previous studies (11, 36, 37). Moreover, notably, traction of the upper pole of the thyroid may change the position and classification of the EBSLN, increasing the proportion of type 2b. Due to the close proximity of the EBSLN to the STA, the high proportion of type 2 EBSLNs also indicates that it is meaningful to identify the EBSLN by routine exposure to reduce the risk of injury.

It was our experience that the STA was unsuitable as a landmark to find the EBSLN intraoperatively, and there were many variations both in the EBSLN and STA (38). Different from cadaveric anatomy, the position of the sternal thyroid muscle and superior posterior thyroid EBSLN and their relationship with the superior artery change during surgery, especially when the thyroid lobe is pulled. This also explains why the intraoperative exposure rate of the EBSLN is generally lower than that of cadaveric studies (39). Moreover, the possible small branches of the superior thyroid artery may bleed and affect the exposure.

According to a previous study (11), we used the insertion of the sternothyroid muscle as a reliable landmark to localize the NEP of the EBSLN. The NEP consistently lies close to the sternothyroid muscle insertion onto the oblique line and does not vary with the direction of pull on the lobe; there is not a bothersome ligament or vessel nearby. We look for the EBSLN in this area visually with the video assistance of a wide field of vision and magnification. In our study, the NEP was approximately 1.18 mm from the sternothyroid insertion and 5–15 mm from the anterior border of the muscle.

After finding the NEP, the nerve was readily traced superiorly to the intersection with the STA. With identification of the EBSLN, the superior thyroid vessels were adequately dissected for safe ligation and division. Our strategy greatly improved the exposure and ease of dissection of the EBSLN, thus reducing the chance of postoperative voice problems. For experienced surgeons with an understanding of local anatomy, this process can be accurate and fast.

The present study has some limitations. First, the sample size of a single institution was not sufficient. For the purpose of controlling postoperative sound function and operation time, we only included patients with unilateral lobectomy, and a large-scale multicenter study with a wider range of patients is needed to further compare the clinical effects. Second, we performed intraoperative visualization, IONM, voice assessments and laryngoscope evaluation to analyze the rates of exposure and injury but did not conduct CTM electroneuromyography (EMG), which is the most accurate tool for diagnosing abnormal EBSLN conductivity. For IONM, we only used cricothyroid muscle contraction to evaluate neural integrity. Due to the limitation of medical insurance costs, we cannot use the intratracheal electrode and cricothyroid electrode to quantitatively measure the EMG signal, which makes us unable to predict postoperative nerve function according to the change in signal strength. Third, the present study did not involve patients with large goiters, short necks or obesity. We also excluded patients with ETE of the upper pole; thus, the 100% exposure rate in the VAS group and 0% injury rate in this study may not accurately reflect the real status of clinical practice. We will conduct further research on these topics in follow-up studies.

## 5. Conclusion

In this study, we used a video-assisted method to fully expose the upper pole of the thyroid. Without increasing muscle injury or increasing the extent of surgery, a good visual exposure rate of the EBSLN was obtained, which was helpful for identifying and protecting the EBSLN during thyroidectomy. This method also represents a new strategy for exposing the EBSLN in patients when IONM cannot be applied. We showed that the EBSLN entry point was localized within 2 mm from the sternothyroid muscle insertion and 5–15 mm from the anterior border of the sternothyroid muscle. A detailed understanding of the anatomical variation in the EBSLN is helpful for finding the EBSLN in the cricothyroid space to avoid the chance of EBSLN injury. In the future, we intend to make the operation simpler and more feasible by using a smaller wireless lens that can be magnetically attached to the hook.

## Data Availability

The original contributions presented in the study are included in the article/Supplementary Material, further inquiries can be directed to the corresponding author/s.
